# Vascularization of Cell-Laden Microfibres by Femtosecond Laser Processing

**DOI:** 10.3390/ijms23126636

**Published:** 2022-06-14

**Authors:** Isabel Verit, Laura Gemini, Julie Preterre, Pierre Pfirmann, Hugo Bakis, Jean-Christophe Fricain, Rainer Kling, Claire Rigothier

**Affiliations:** 1 ALPhANOV, Institut d’Optique d’Aquitaine, Rue François Mitterrand, 33400 Talence, France; isabel.verit@gmail.com (I.V.); rainer.kling@alphanov.com (R.K.); 2 Department of Tissue Bioengineering, Université de Bordeaux, Rue François Mitterrand, 33076 Bordeaux, France; julie.preterre@chu-bordeaux.fr (J.P.); pierre.pfirmann@chu-bordeaux.fr (P.P.); hugo.bakis@chu-bordeaux.fr (H.B.); jean-christophe.fricain@inserm.fr (J.-C.F.); claire.rigothier@chu-bordeaux.fr (C.R.); 3 Department of Tissue Bioengineering, Institut National de la Santé et de la Recherche Médicale (INSERM) U1026, 33076 Bordeaux, France; 4 Service de Néphrologie, Transplantation, Dialyse et Aphérèse, Centre Hospitalier Universitaire de Bordeaux, Place Amélie Raba Léon, 33000 Bordeaux, France; 5 Service d’Odontologie et de Santé Buccale, Centre Hospitalier Universitaire de Bordeaux, Place Amélie Raba Léon, 33000 Bordeaux, France

**Keywords:** vascularization, femtosecond laser, cell-laden microfibre, tissue engineering

## Abstract

To face the increasing demand for organ transplantation, currently the development of tissue engineering appears as the best opportunity to effectively regenerate functional tissues and organs. However, these approaches still face the lack of an efficient method to produce an efficient vascularization system. To answer these issues, the formation of an intra-volume channel within a three-dimensional, scaffold free, mature, and cell-covered collagen microfibre is here investigated through laser-induced cavitation. An intra-volume channel was formed upon irradiation with a near-infrared, femtosecond laser beam, focused with a high numerical aperture lens. The laser beam directly crossed the surface of a dense and living-cell bilayer and was focused behind the bilayer to induce channel formation in the hydrogel core while preserving the cell bilayer. Channel formation was assessed through confocal microscopy. Channel generation inside the hydrogel core was enhanced by the formation of voluminous cavitation bubbles with a lifetime longer than 30 s, which also improved intra-volume channel durability. Twenty-four hours after laser processing, cellular viability dropped due to a lack of sufficient hydration for processing longer than 10 min. However, the processing automation could drastically reduce the cellular mortality, this way enabling the formation of hollowed microfibres with a high density of living-cell outer bilayer.

## 1. Introduction

The recent expansion of tissue engineering allowed the development of innovative techniques to create tissue substitutes as a response to the organ shortage [1]. However, the lack of an existing and efficient vascularization system prevents those substitutes from being implanted in the host body [2,3]. Among existing vascularization strategies, some studies mimicked angiogenesis and vasculogenesis mechanisms to induce vessels’ formation, growth, and expansion inside the host body [4,5,6]. While promising, the development of a mature blood vessel network remains time-consuming and inadequately provides oxygen and nutrients to the host’s cells shortly after the surgery. Another approach enabling a more rapid vascularization of the substitutes after implantation is to directly create three-dimensional (3D) micro blood-vessel substitutes and integrate them into the tissue substitutes [7,8,9]. Based on engineering techniques such as stereolithography [10,11], microfluidic [6,12], or bioprinting [13,14], some studies highlight the generation of 2D capillary models or 3D scaffold-embedded microchannels. However, none of these techniques allow the formation of a mature, scaffold-free, and perfusable microcapillary in 3D, conditions required to be implanted and to provide an efficient vascularization.

Here, we propose a method to create an intra-volume channel within 3D, scaffold-free, mature, and cell-covered hydrogel microfibres inspired by industrial laser processing techniques applied to transparent materials. Primarily, a microfibre was produced from a collagen hydrogel core covered by a dense and living-cell outer bilayer. Secondly, a channel was processed inside the collagen core with a near-infrared (NIR, Stockholm, Sweden), femtosecond (fs) laser source. It is well known that the use of tightly focused ultrashort laser pulses induces localized damages in the bulk of transparent materials via non-linear absorption while minimizing thermal effects in the interaction zone [15]. Indeed, NIR laser beams allow working in the tissue biological window where the laser light is less scattered or absorbed by the material [16].

Data on laser-hydrogel interaction have been previously reported by the same group, Verit et al. [17]. In addition, several studies report about the generation of intra-volume channels within the volume of cell-embedded hydrogels. Applegate et al. generated 3D structures within the volume of a silk-based hydrogel with ultrashort laser pulses (100 fs) at a wavelength of 810 nm. Here, silk hydrogel was chosen for its transparency to the 810 nm wavelength enabling the creation of voids at 1 cm depth. The use of a pulse repetition rate of 80 MHz generated a strong thermal accumulation at the focal point disrupting silk fibres and leading to the apparition of a cavity inside the hydrogel [18]. To generate channels within collagen hydrogel, Hribar et al. doped the hydrogel with gold nanorods, creating a local absorption pick at 800 nm. Using 100 fs laser pulses with only a few nJ and at a repetition rate of 80 MHz, nanorods efficiently absorbed the laser beam which was then converted into heat disrupting collagen fibres and generating channels [19]. Using UV laser light (355 nm) and nanosecond pulses (1 ns) at a repetition rate of 100 Hz, Brandenberg et al. generated channels in the bulk of a bovine collagen hydrogel. The use of UV light and nanosecond pulses leads to channels at shallower depths and greater widths with reduced machining accuracy [20]. The common characteristic to these three studies and the majority of studies found in the literature is the ability to preserve cellular viability right after hydrogel laser machining [19] or up to 4 h after laser interaction [18]. However, the good cellular viability after laser processing may be explained by the small number of embedded cells scattered throughout the material’s volume; when the beam goes through the hydrogel to generate an intra-volume channel, only a few cells happen to interact with it. In addition, Zeigler et al. have shown that a decay in cellular viability could appear up to 12 h after laser processing, whereas cellular viability tracking in the previously mentioned studies was limited to a few hours only after processing [21].

The uniqueness of this work lies in the generation of channels within the volume of collagen hydrogel microfibres covered by a high-density bilayer of living cells, in order to closely mimic a real glomerular microfibre system. During laser processing, the laser beam directly crossed the surface cell bilayer before being focused in the hydrogel volume to create cavities. Cellular viability was evaluated using Live/Dead^®^ assays 24 h after laser processing to assess the influence of direct laser interaction on cell preservation. The experimental approach employed in this work is based on a previous study by the same group on intra-volume fs laser processing of cell-free gelatin hydrogel blocks where cavitation phenomena induced by a tightly focused fs laser in the hydrogel volume were investigated [17]. It was observed that after one laser pulse, a cavitation bubble appeared at the focal point, rapidly grew to reach its maximum size, and then slowly shrank in volume affecting the gelatin hydrogel structure. Finally, it was found that channel formation was promoted when a low number of large volume cavitation bubbles characterized by a slow shrinking rate was observed in the gelatin hydrogel volume after the laser processing. This behavior was enhanced when processing at a depth of the focal point of 100 μm, at fluence higher than the cavitation threshold value of 24.5 J·cm^−2^, at low repetition rate, and with a strong spatial overlap between two successive laser pulses. These results were considered as a starting point for this study, where the number of cavitation bubbles formed, their maximal size, and their shrinking rate depended on laser parameters and directly affected channel formation and cellular viability in the microfibres.

## 2. Results

### 2.1. Channel Formation within the Collagen Hydrogel Core

#### 2.1.1. Variation of the Depth of FOCUS D

**Figure 1:**
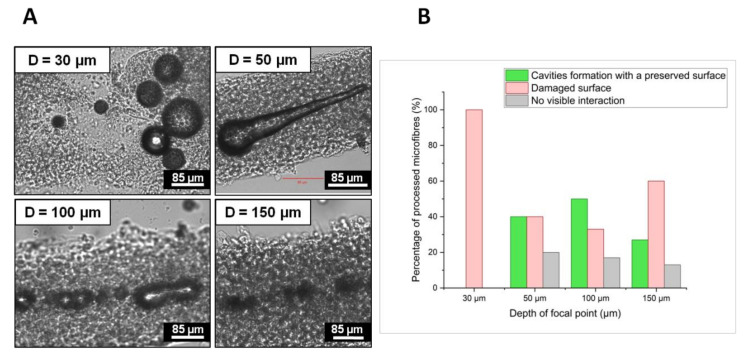
(**A**) CCD camera images of cavitation bubbles recorded 5s after laser processing and observed inside four different microfibres processed with the same laser parameter and four different depths of focus: D = 30 μm, 50 μm, 100 μm, and 150 μm. Cavitation bubbles were imaged in the collagen hydrogel core at the processing depth. Fixed process parameters: λ = 1030 nm; F = 22.1 J/cm^2^; R.R. = 50 kHz; O.L. = 99.7%; two consecutive laser passes. (**B**) Evolution of the microfibre number (%) with respect to the type of laser-induced modifications observed 24h after laser processing for different depths of focus.

Figure 1A presents cavitation bubbles recorded by CCD camera right after laser processing at different depths D of the focal point inside the hydrogel core of four different representative microfibres. Figure 1B shows a statistic representation of two different types of laser-induced modifications observed 24 h after laser processing at different depths of focus:

- Red bars represent the percentage of microfibres where surface damages such as cell bilayer ablation, perforation, or complete destruction of the microfibre structure were observed;
- Green bars represent the percentage of microfibres in which cavities or channel formation was detected without the presence of surface damage;
- Grey bars are added to account for the percentage of microfibres where no visible interaction was observed;

For each depth of focus, bars indicated the percentage of microfibres calculated from the analyses of 20 different microfibres, except for D = 30 μm where only four microfibres were retrieved after laser processing. Two different and competitive phenomena arise; at shallow depths, most of the laser energy reaches the focal point within the hydrogel and results in the formation of large cavitation bubbles, which remain trapped longer within the hydrogel core. Their size decreases and their lifetime shortens when the laser is focused deeper in the core, given that a larger part of the laser light is absorbed or scattered before reaching the focal point. For instance, at D = 150 μm, the bubble average diameter was about 18.9 μm and bubbles disappeared completely in a few seconds. The average diameter increased to 32.8 μm and 54.0 μm, with a lifetime ranging from 30 s to a few minutes for D = 100 μm and over 30 min for D = 50 μm.

**Figure 2:**
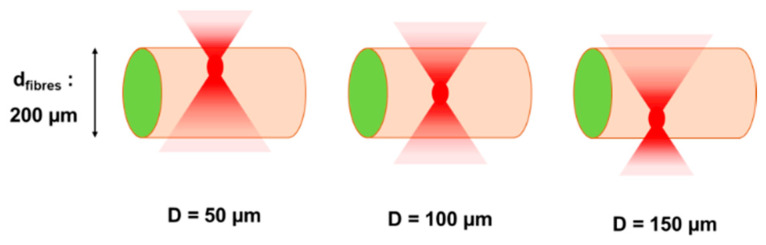
A schematic illustration of focal point positions for different depth of focus values inside a 200 µm diameter microfibre. Red gradient cones map the laser fluence gradient before and after focalization. The further away from the focal volume, the lower the laser fluence.

The second observation that can be deduced in connection to a variation of the depth of focus is related to the cell bilayer wall damages. It was observed that the larger the cavitation bubbles and the closer they were to the cell wall, the more microfibres were damaged on the surface. Figure 2 illustrates how the position of the focal spot inside the volume of a 200 μm diameter microfibre may influence the cell viability. Red spots correspond to the focal voxel and red gradient cones map to the laser fluence gradient before and after the focal spot; the laser energy per unit of irradiated surface is lower moving away from the focal voxel. As a consequence, for a given fluence value, when the focal spot is localized close to the cell wall, the laser fluence outside the focal cone can exceed the cell wall damage threshold. On the contrary, when the focal spot is located closer to the center of the microfibre, the fluence value on the cell wall might be sufficiently low to avoid a strong interaction of the material with the laser. Thus, for D = 100 μm, when the laser beam was focused approximately at the center of the microfibre, cavitation bubbles are sufficiently large to generate a channel and distant from the cell wall to avoid surface damages due to the bubble expansion mechanism. The probability of creating a channel without damaging the cell wall was 50% at D = 100 μm from the microfibre surface and decreased to 27% and 40% for D = 140 μm and D = 50 μm, respectively (Figure 1B). For D = 30 μm, cavitation bubbles were too voluminous and too close to the cell wall, and microfibre disruption was observed in all processed microfibres (Figure 1A).

#### 2.1.2. Variation of the Laser Fluence F

**Figure 3:**
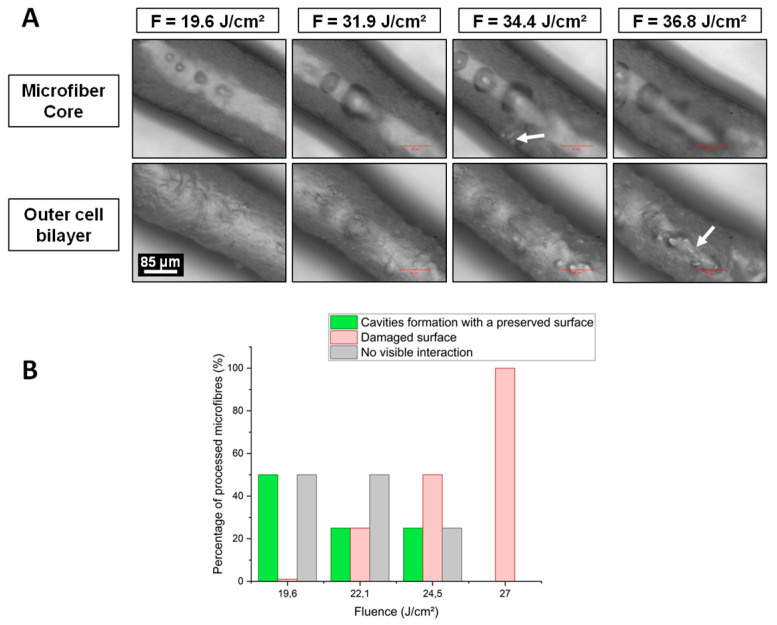
(**A**) One microfibre processed at four different fluence values: F = 19.6; 31.9; 34.4, and 36.8 J·cm^−2^, Top: microfibre core, Bottom: outer cell bilayer. Cavitation bubbles were imaged within the collagen hydrogel at the processing depth (90 µm). Fixed parameters: λ = 1030 nm; D = 90 µm; R.R. = 50 kHz; O.L. = 99.7%; 1 laser pass. White arrows indicate significant cell wall damages. (**B**) Evolution of the microfibres number (%) with respect to the type of laser-induced modifications observed 24h after laser processing for different fluence values. A schematic illustration of focal point positions for different depth of focus values inside a 200 µm diameter microfibre. Red gradient cones map the laser fluence gradient before and after focalization. The further away from the focal volume, the lower the laser fluence.

Figure 3A shows laser-induced cavitation bubbles images recorder by CCD camera right after laser processing in the volume of the same microfibre at different values of laser fluence. Figure 3B presents the percentage of microfibres for two different types of laser-induced modifications observed 24 h later with respect to the variation of the laser fluence. Cavities or intra-volume channel formation inside microfibres without surface damage is shown in green colored bars. Red bars represent microfibres whose surface has been left damaged by laser processing. Each average value was calculated from four different microfibres.

Increasing the laser fluence while keeping all other processing parameters fixed led to increasingly larger cavitation bubbles, which remained trapped inside the microfibres for longer time. For instance, at F = 19.6 J·cm^−2^, the bubble mean diameter was about 25 µm and increased to 85 µm at F = 31.9 J·cm^−2^, and the cell wall was not damaged. However, as the fluence continued to increase, bubbles became large enough to pierce the cell wall. For F = 34.4 J·cm^−2^, the white arrow (Figure 3A) shows a hole in the wall created by a very large cavitation bubble, and at F = 36.8 J·cm^−2^, the presence of a sharp line demonstrates the cell wall ablation (second white arrow in Figure 3A).

A decrease in fluence reduced the laser-induced surface damages and optimized channel and cavities formation in the hydrogel core of microfibres. Optimal fluence was reached at F = 19.6 J·cm^−2^ where 50% of microfibres had an intra-volume channel and a preserved cell wall (Figure 3B). It is important to note that below F = 19.6 J·cm^−2^, neither cavitation bubbles nor channels formation could be observed.

### 2.2. Cellular Viability

#### 2.2.1. Effect of Thermal Accumulation

**Figure 4:**
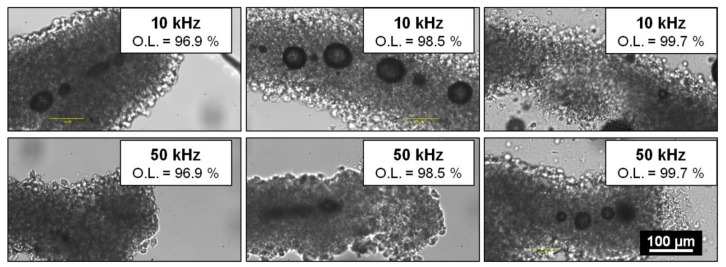
Cavitation bubble behavior 5s after laser processing within different microfibres with respect to the repetition rate: 10 kHz (top) and 50 kHz (bottom) and the overlap: 96.9% (left), 98.5% (middle), and 99.7% (right). Fixed parameters: λ = 1030 nm; D = 100 µm; F = 24.5 J·cm^−2^; 1 laser pass.

According to literature, laser-induced thermal load is a relevant parameter in cell preservation during nano-processing of cells and tissues [21,22,23]. During laser processing, heat accumulation can be modulated by varying several process parameters, such as the laser fluence, the repetition rate, or the spatial overlap between two successive pulses. For a given laser pulse energy, an increase in the repetition rate for a given overlap or an increase in the overlap for a given repetition rate results in higher thermal loads in the material volume. Figure 4 presents CCD camera images of cavitation bubbles right after laser interaction for different repetition rates and spatial overlap values. For each set of parameters, two microfibres were processed for sake of repeatability.

The observed cavitation bubble behavior was different when the heat accumulation increased by raising either the repetition rate or the overlap (Figure 4). An increase in the spatial overall between successive laser pulses led to a lower number of larger cavitation characterized by a longer lifetime. On the contrary, when the laser repetition rate was raised, cavitation bubbles were more numerous, smaller, and characterized by a shorter lifetime. For instance, at 10 kHz and an overlap of 96.9%, the bubble mean diameter was about 22.5 μm with a lifetime ranging from 1 to 30 min. The mean diameter reached 54.6 µm with a lifetime of up to 1 h and 30 min when the process was carried with a higher overlap of O.L. = 98.5% for the same repetition rate. For higher values of spatial overlap (O.L. = 99.7%), bubbles exerted stronger mechanical pressure over the cell wall leading to microfibre disruption. Conversely, for a given overlap value of O.L. = 96.9%, the mean diameter decreased from 22.5 µm at 10 kHz to 11.5 µm at 50 kHz with a lifetime of less than 10 s.

To summarize, thermal accumulation played a role in cavitation bubble behavior but did not systematically affect cellular viability. No correlation was detected between applied laser parameters and cell death rate. However, significant mortality was observed for the majority of microfibres 24 h after laser processing by using a Live/Dead^®^ kit.

#### 2.2.2. Effect of Microfibre Hydration

**Figure 5:**
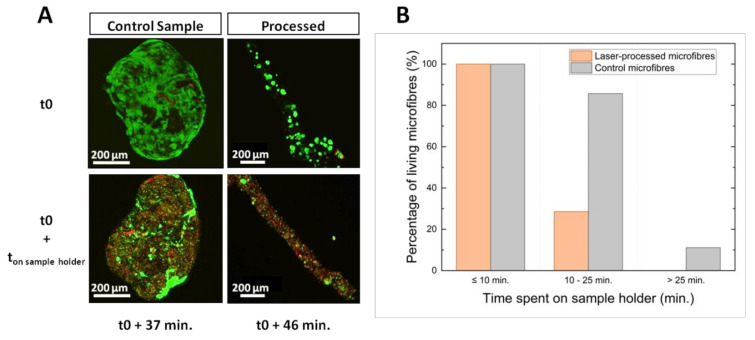
Evolution of cellular viability according to microfibre hydration. (**A**) Confocal microscope images of two microfibres (one control microfibre and one laser-processed microfibre) stored in culture medium (t0) and the same microfibres 37 and 46 min after the culture medium was removed. Only the processed microfibre received laser treatment. (**B**) Histogram showing the percentage of microfibres still alive after 24 h as a function of the time spent on the sample holder with only a small amount of culture medium for the group of laser-processed microfibres (orange) and the group of control microfibres (grey).

To understand the origin of cell mortality, the cellular viability evolution as a function of microfibre hydration was investigated for one laser-processed microfibre and a control microfibre, which was handled the same way except for the laser processing step. These microfibres were firstly placed in an agarose-coated dish. Just before laser processing, the culture medium was almost completely removed to avoid dispersion of the laser energy, and consequently a process of dehydration of the microfibres was induced. One microfibre was then laser processed and analyzed 46 min after the removal of culture medium while the second was left in the same environment and analyzed 36 min later. The time between the removal of the culture medium (t0) and the analyses is defined as ton sample holder. Results are presented in Figure 5.

Cellular viability of the two microfibres is presented in Figure 5A. Figure 5B shows the percentage of living microfibre as a function of ton sample holder. Orange and grey bars indicate cellular viability for microfibres of the laser-treated group and the control group which did not receive any laser treatment, respectively.

**Figure 6:**
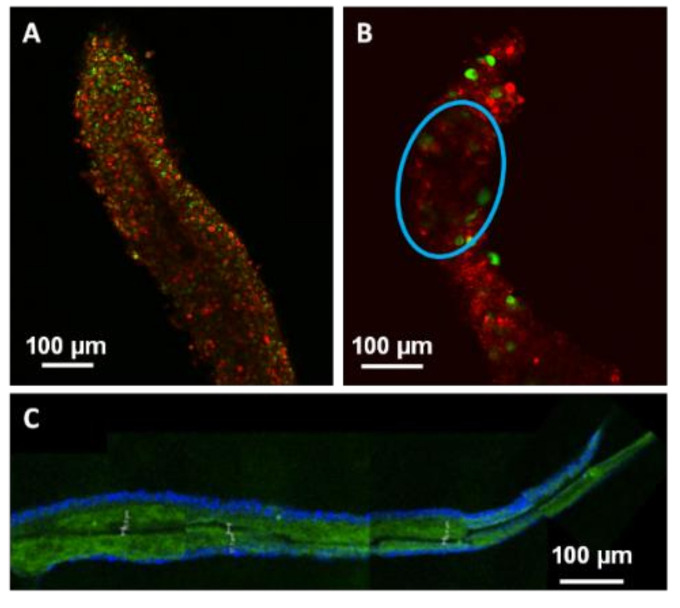
Confocal microscope images showing laser-induced modifications in three different types of microfibres. (**A**) Sharp channel inside a dehydrated microfibre after spending 41 min on a sample holder. (**B**) Attenuation (blue circle) of signal density inside a hydrated microfibre after 9 min spent on a sample holder. After being marked with Live/Dead^®^ ki, A and B colors were modified to highlight laser interaction effects. (**C**) Sharp channel inside a PFA-fixed microfibre, green: collagen-Fluorescein-5-isothiocyanate (FITC), blue: DAPI staining.

**Figure 7:**
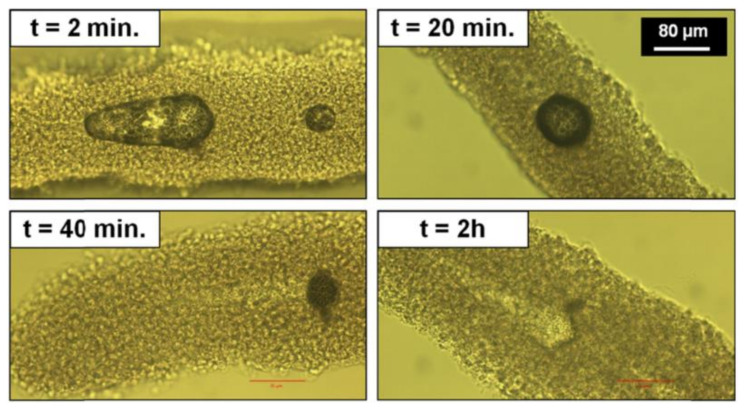
Temporal evolution of a laser-induced cavitation bubble inside the hydrogel core of a single microfibre. Inserts indicate the time elapsed since the end of laser processing.

The cell death induced by laser processing was not significantly different from the cell viability observed for the group of non-processed microfibres (Figure 5A). The impact on cellular viability could not be only explained by the laser processing effect but it was probably due to the microfibre conditioning during laser treatment. Indeed, as represented in Figure 5B, cellular viability decreased with an increase of the period spent on the sample holder. All microfibres, whether laser processed or not, remained viable after less than 10 min on the sample holder. As the dehydration period increased, cellular viability began to drop and was accentuated for laser-treated microfibres. Between 10 and 25 min, the 86% of the microfibres of the control group was viable against the 29% only of the processed ones. Beyond 25 min, all processed microfibres were dead while 11% of the controls were still viable. Therefore, the loss of viability observed on laser-treated microfibres was partly explained by the water stress endured by the cells due to a prolonged time spent on the sample holder combined with the stress due to the laser processing. Hydration influenced cellular viability and the laser processing quality. Figure 6 presents laser-induced modifications in three different types of microfibres. The first microfibre (A) was completely dehydrated after being kept with a low hydration supply for more than 25 min. The second microfibre (B) remained hydrated after staying only 9 min on the sample holder. The colors of microfibres A and B were modified for contrast enhancement to better visualize the channel formation. Finally, the third one (C) was PFA-fixed prior to laser processing. A DAPI staining was applied to highlight the cell bilayer. A temporal evolution from laser-induced bubble to channel formation in a dehydrated microfibre is also depicted in Figure 7.

While a change in hydration did not cause any change in the behavior of cavitation bubbles (as for comparison with Figure 4), it appeared to directly influence the channel formation 24 h after laser processing. When microfibres were dehydrated causing a significant drop in cellular viability, channels were thin and sharp (Figure 6A). In contrast, within hydrated microfibres, only an attenuation of the red signal from collagen matrix was detected (Figure 6B).

For dehydrated microfibres, channel creation was easily detected by studying the disappearance of cavitation bubbles under an optical microscope. Figure 7 shows the temporal evolution of a cavitation bubble between 2 min and 2 h after laser interaction. The bubble gradually shrank until it disappeared completely, leaving a sharp channel in its wake. This phenomenon was visible for large cavitation bubbles, which took longer than 30 min to disappear. Channel formation within dehydrated microfibres was similar to that of PFA-fixed microfibres (Figure 6C) while cavitation bubbles in hydrated microfibres disappeared with no sharp channel formation.

## 3. Discussion

This work aims to identify a narrow range of laser parameters as well as the best experimental conditions allowing the creation of a fully formed channel within the collagen core of microfibres without damage to the outer dense cell bilayer. Two new challenges were identified with respect to our previous works [17,24]: firstly, the identification of an efficient laser parameter window allowing channel formation in the hydrogel core and secondly, the preservation of the cellular viability of this high-density living cell bilayer.

To achieve these goals, the optimal laser parameters were determined from previous observations that channel generation inside a hydrogel was enhanced by the formation of voluminous cavitation bubbles with a lifetime from a few minutes to a few hours before collapse [17]. This type of bubbles was generated inside a gelatin hydrogel because of the similarity of its optical properties to those of collagen hydrogels [25]. The best set of process parameters was identified at a depth D = 100 µm, a fluence F = 24.5 J·cm^−2^, a repetition rate R.R. = 10 kHz and an overlap O.L. ≤ 98.5%. Under these conditions, material damages due to heat dispersion were spatially limited to the channel close vicinity. Moreover, cavitation bubbles were the farthest from the outer cell bilayer thus minimizing mechanical pressure applied on cells. If the focal point was positioned too close to the cell wall (D < 100 µm), fluence value at the microfibre surface could exceed the cell bilayer damage threshold leading to a cell viability drop or eventually the complete destruction of the microfibre structure. On the other hand, for depths greater than D = 100 µm, laser energy was likely absorbed and dispersed by the high-water content collagen, leading to a decrease of the volume of cavitation bubbles and their probability to generate channels.

By varying fluence values at D = 100 µm, the volume of cavitation bubbles and their respective lifetime could be enhanced to promote channel generation. While a fluence lower than F = 19.6 J·cm^−2^ did not create any cavitation bubble, highest fluence values generated non-linear effects directly on the microfibre outer cell bilayer. Above F = 36.8 J·cm^−2^, the fluence value at the cell wall likely exceeded the cell bilayer damage threshold, altering the laser-irradiated cells.

In literature, thermal accumulation during laser processing is a relevant factor especially when it comes to biological systems where cellular viability preservation is essential [21,23,26]. The thermal load could be reduced by decreasing either the repetition rate or the spatial overlap between two successive pulses. The results presented above showed that a decrease of thermal accumulation directly influenced the behavior of cavitation bubbles during laser interaction. However even when cavitation bubbles with a diameter smaller than the average width of the collagen core were created far enough from the cell wall, no cellular viability improvement was observed. It is important to note that a strong cellular viability drop was generally observed independently of the applied laser parameters and related thermal accumulation effects.

Live/Dead^®^ assays revealed that the viability drop was accentuated by microfibre dehydration. Only a minimal amount of culture medium was used during the laser processing to prevent culture medium from dispersing the laser energy. In this environment, cell damage was negligible for about 10 min. Afterwards, cellular viability decreased quickly due to stress related to dehydration and laser processing. These results show that a good preservation of cellular viability might be possible by reducing the total processing time below 10 min. Reducing this time would be achievable by a process automation involving the detection of the microfibre center and the calculation of the laser trajectory via a synchronization of the CCD camera with the stage control software.

Cell hydration also affected the laser processing quality as observed by confocal microscopy 24 h after process. When microfibres were non-viable due to insufficient hydration, laser processing led to the formation of a sharp channel within the dried collagen core. These channels were generated in a similar way than the ones observed inside PFA-fixed microfibres or in synthetic hydrogel [27]. On the other hand, when microfibres were viable due to sufficient hydration, channel detection was difficult. Either no channel or only a slight attenuation of the autofluorescence signal from the hydrogel core was detected. This observation could be explained by the fact that the collagen of hydrated fibres might be able to reabsorb water after bubble collapse due to its high water content. This way, the channel would slowly be clogged after the laser interaction. To preserve channel durability, processed microfibres should be immediately perfused after laser processing. Moreover, the presence of an internal flow might allow complete cell differentiation of the outer cell bilayer within this dynamic and 3D environment [28,29,30,31].

## 4. Materials and Methods

Collagen hydrogel microfibres were composed of a hydrogel core made of collagen type I and a cell bilayer with glomerular endothelial cells (GECs) on the inner side and podocytes on the outer side.

### 4.1. Cell Culture

Outer cell bilayer was composed of GECs and podocyte cells immortalized by temperature sensitive gene SV40. They were cultured according to the protocol by Flegeau et al. [22]. Both cell lines proliferated at 33 °C and differentiated at 37 °C.

### 4.2. Living-Cell Microfibres Manufacture

Firstly, a shell of 2% reticulated alginate was made around a 350 µm diameter polycarbonate capillary tube (Paradigm Optics, Vancouver, WA, USA) by soaking it alternatively into sodium alginate (FMC BioPolymer, Philadelphia, PA, USA) and calcium chloride (Sigma Aldrich, Burlington, MA, USA) baths. The polycarbonate tube was then removed, and a solution containing type I collagen (Corning Lab, Corning, NY, USA) and GECs was simultaneously injected inside the core of the alginate shell. Since collagen solution is liquid below 4 °C, the injection was performed at cold temperature. Alginate shells were then manually removed after 18 h incubated at 33 °C. Five additional days were needed at 37 °C in order to obtain a full differentiation of GECs and their migration to the outer part of hydrogel microfibres. Secondly, podocytes were seeded over GECs, along microfibres, in order to obtain a cell bilayer covering the core of collagen hydrogel. Fourteen days after seeding, podocytes were differentiated. Cell number (35.10^6^/mL) and collagen concentrations (5 mg/mL) required to create collagen hydrogel microfibres was determined in the previous study of Flegeau et al. [22].

### 4.3. PFA-Fixed Microfibres Manufacture

PFA-fixed microfibres were created following the previous protocol [22]. However, collagen type I-FITC (Sigma Aldrich, Burlington, MA, USA) was used instead of the Corning Lab collagen I. Then, microfibres were washed with PBS 1X and sank in a solution of 4% paraformaldehyde (PFA) at 4 °C. After 30 min, they were rinsed three times with PBS 1X. PFA-fixed microfibres were either immediately laser processed or stored in 12-well culture plates with PBS 1X at 4 °C.

### 4.4. Femtosecond Laser Processing Set-Up

Laser processing of microfibres was carried with a 350 fs laser source (Satsuma HP3 from Amplitude System, Pessac, France) running at 1030 nm central wavelength. The laser pulse energy was adjusted by sending the Gaussian linearly polarized laser beam through a polarizer and a half-wave plate. A beam expander of a factor 3 combined with a 0.4 numerical aperture microscope objective (Seiwa Optical, Seiwa Optical Europe GmbH, Frankfurt, Germany) ensured a strong focalization into the bulk of the material with a spot diameter at the focal point of about 3 µm. Sample holders were placed on computer-controlled XY translation stages (ALIO, Los Angeles, CA, USA) while the vertical position of the focusing objective was controlled by a Z translation stage (ALIO, Los Angeles, CA, USA) to precisely determine the focus position. A more detailed description of the experimental set-up used for processing and imaging the microfibres is provided in Verit et al. [17].

### 4.5. Sample Conditioning before Processing

Microfibres (length in the range 0.5–1 cm) were laid on a 35 mm diameter Petri dish coated with a solution of 2% agarose with a drop of culture medium. Just before laser processing, the culture medium was removed until the microfibres adhered to the agarose coating, providing a minimal hydration for the cells during processing while avoiding possible absorption and scattering from the culture medium itself. The preparation of microfibres was estimated roughly at 1 h. 

### 4.6. Laser Micromachining Process

Sample holders containing microfibres were placed on the XY translation stages. To generate channels within the collagen core of microfibres, straight lines were processed by the laser beam following the natural and tortuous shape of the microfibre by moving the XY translation stages at a defined translational speed, while keeping the position of the laser beam fixed. Several processing parameters were varied in order to evaluate their influence on the formation of channels within the microfibres: depth of the focal point within the microfibre (D), laser fluence (F), laser repetition rate (R.R.), and spatial overlap between two successive laser pulses (O.L.). The depth of the focal point within the material volume was ranging from D = 30 µm to D = 150 µm, where the reference D = 0 µm was considered to be the outer surface of the microfibre. The fluence ranged from F = 19.6 J·cm^−2^ to F = 36.8 J·cm^−2^. Two repetition rate values were investigated: R.R. = 10 kHz and R.R. = 50 kHz while the spatial overlap between successive laser pulses was varied by changing the speed v of the XY translation stages from v = 0.1 mm·s^−1^ to v = 5 mm·s^−1^ in order to study the influence of the following overlap values (O.L.): 96.9%, 98.5%, and 99.7%. While samples stayed on the laser processing station for just a few minutes for the treatment, the time for their preparation and handling just before and after the laser processing was estimated to up to 15 min. For each combination of laser parameters, experiments were repeated twice for the sake of repeatability. For all collected data, std <5% was obtained. 

### 4.7. Characterization of Laser-Processed Living Cell Microfibres and Cell Viability Assessment

For each set of laser parameters, the microfibres were characterized at two different moments in time: immediately after and 24 h after laser interaction. After laser processing, processed areas were imaged via a CCD camera acA2000 (Basler, with a 0.4 N.A.) whose image plane followed the laser focal point during laser interaction. Channel formation and cellular viability were assessed 24 h later by confocal microscopy (Leica, Wetzlar, Germany) with a Live/Dead^®^ cytotoxicity assay (Life Technologies, Carlsbad, CA, USA).

### 4.8. Characterization of Laser-Processed PFA-Fixed Microfibres

A DAPI solution was diluted at 1/5000 in a PBS 1X solution and applied on laser-processed PFA-fixed microfibres during 10 min in dark environment, then rinsed three times with distilled water. DAPI-labeled cell nucleus appeared in blue color in confocal microscope images (Leica) while collagen FITC appeared in green color.

## 5. Conclusions

The goal of this work was to develop a 3D blood microcapillary model by femtosecond laser processing. Whereas intra-volume channels are commonly created inside cell-free hydrogel or with a low density of embedded cells scattered through the whole material volume [18,19,20], here, the laser processed system is upgraded to mimic more closely a real microcapillary structure, where hydrogel microfibres are enveloped by a high cellular density which strongly affects the laser processing outcomes. Moreover, the preservation of the cellular viability is assessed up to several hours after the laser processing. Channel processing was realized with a 350 fs, 1030 nm laser source strongly focused by a high numerical aperture objective. Channel formation, due to laser-induced cavitation in the hydrogel core, was optimized by processing at a focal depth of D = 100 µm, a laser fluence of F = 19.6 J·cm^−2^, an overlap between O.L. = 96.9% and O.L. = 99.7%, and a repetition rate between R.R. = 10 kHz and R.R. = 50 kHz. These conditions allowed the generation of voluminous cavitation bubbles with a lifetime longer than 30 s, which was demonstrated to improve intra-volume channel durability. However, laser processing resulted in a decrease of cellular viability regardless the specific laser parameters applied for the processing. This viability drop was explained by a lack of sufficient hydration necessary to obtain a successful processing. Cellular mortality could be reduced drastically by reducing the dehydration stress through automation of the microfibre center detection and calculation of the laser trajectory. The last issue to consider concerns the sustainability of the channel created inside fully hydrated microfibres, which could be addressed by the perfusion of processed microcapillaries immediately after laser processing. To extend the exploitation of this technique to the generation of full 3D fibrous scaffolds for vascularization of thick constructs, the combination of successive manufacturing steps of laser-induced vascularization/issue generation with the employment of an automated six-axis system, which allows for laser treatment of 3D parts, could be eventually considered in a single manufacturing environment. 

## Data Availability

Not applicable.
